# Molecular targets for PDE inhibitor-mediated improvement of cardiac dysfunction in the mdx mouse?

**DOI:** 10.1186/1471-2210-11-S1-O20

**Published:** 2011-08-01

**Authors:** Candace M Adamo, Dao-Fu Dai, Justin M Percival, Elina Minami, Monte S Willis, Enrico Patrucco, Sergei D Rybalkin, Stanley C Froehner, Joseph A Beavo

**Affiliations:** 1 University of Washington, Department of Pharmacology, USA

## 

Recent results show that the classic PDE5 inhibitor, Viagra® (sildenafil), can ameliorate much of the cardiac pathology resulting from several different forms of cardiac damage. These results have elicited great interest in the use of this drug as a therapeutic agent, particularly for treatment of diastolic cardiac dysfunction. In fact several clinical trials are now enrolling and one published trial shows promising results. Unfortunately, it is not clear how this drug works at a molecular level to improve cardiac function and not even clear what the initial molecular target(s) are for Viagra® in any of these studies. Recent data from our group and others strongly suggest that sildenafil, a “classical” PDE5 inhibitor, ameliorates much of the diastolic dysfunction seen in older mdx mice, animals having a disruption in the dystrophin gene. However, our preliminary data suggests that tadalafil does not cause this effect. Moreover, the usual accepted target for sildenafil, PDE5, is not even expressed in adult mouse cardiomyocyes; but an alternate or additional target, PDE1C is highly expressed in this cell type. Since PDE1C, also can be substantially inhibited by sildenafil (but not by tadalafil) we feel that the most likely molecular target for Viagra® is a direct effect on PDE1C in the cardiomyocyte itself. There also may be additional effects on PDE5, but probably in another cell type. These topics will be discussed in this talk.

